# Functional Study of *TMEM163* Gene Variants Associated with Hypomyelination Leukodystrophy

**DOI:** 10.3390/cells11081285

**Published:** 2022-04-09

**Authors:** Huifang Yan, Shuyan Yang, Yiming Hou, Saima Ali, Adrian Escobar, Kai Gao, Ruoyu Duan, Thomas Kubisiak, Junyu Wang, Yu Zhang, Jiangxi Xiao, Yuwu Jiang, Ting Zhang, Ye Wu, Margit Burmeister, Qiang Wang, Math P. Cuajungco, Jingmin Wang

**Affiliations:** 1 Department of Pediatrics, Peking University First Hospital, Beijing 100034, China; yanhuifang96@bjmu.edu.cn (H.Y.); gaokaipku@hsc.pku.edu.cn (K.G.); ruoyuduan123@pku.edu.cn (R.D.); wjy-pediatrics@bjmu.edu.cn (J.W.); zhangyunihao@bjmu.edu.cn (Y.Z.); jiangyuwu@bjmu.edu.cn (Y.J.); dryewu@bjmu.edu.cn (Y.W.); 2 Joint International Research Center of Translational and Clinical Research, Beijing 100191, China; 3 Beijing Key Laboratory of Molecular Diagnosis and Study on Pediatric Genetic Diseases, Beijing 100034, China; 4 Michigan Neuroscience Institute, University of Michigan, Ann Arbor, MI 48109, USA; kubisiat@umich.edu (T.K.); margit@umich.edu (M.B.); 5 Beijing Municipal Key Laboratory of Child Development and Nutriomics, Capital Institute of Pediatrics, Beijing 100020, China; shuyanyang79@126.com (S.Y.); zhangtingcv@126.com (T.Z.); 6 State Key Laboratory of Membrane Biology, Institute of Zoology, University of Chinese Academy of Sciences, Chinese Academy of Sciences, Beijing 100101, China; houyiming@ioz.ac.cn (Y.H.); qiangwang@ioz.ac.cn (Q.W.); 7 Department of Biological Science, California State University, Fullerton, CA 92831, USA; saimaali@fullerton.edu (S.A.); adrian_escobar@csu.fullerton.edu (A.E.); 8 Department of Radiology, Peking University First Hospital, Beijing 100034, China; xiaojiangxi@bjmu.edu.cn; 9 Key Laboratory for Neuroscience, Ministry of Education/National Health and Family Planning Commission, Peking University, Beijing 100191, China; 10 Departments of Computational Medicine & Bioinformatics, Psychiatry and Human Genetics, University of Michigan, Ann Arbor, MI 48109, USA; 11 Institute for Stem Cell and Regeneration, Chinese Academy of Sciences, Beijing 100101, China; 12 Center for Applied Biotechnology Studies, California State University, Fullerton, CA 92831, USA

**Keywords:** TMEM163 protein, hypomyelination leukodystrophy, zinc efflux transporter

## Abstract

Hypomyelinating leukodystrophies (HLDs) are a rare group of heterogeneously genetic disorders characterized by persistent deficit of myelin observed on magnetic resonance imaging (MRI). To identify a new disease-associated gene of HLD, trio-based whole exome sequencing was performed for unexplained patients with HLD. Functional studies were performed to confirm the phenotypic effect of candidate protein variants. Two de novo heterozygous variants, c.227T>G p.(L76R) or c.227T>C p.(L76P) in *TMEM163* were identified in two unrelated HLD patients. TMEM163 protein is a zinc efflux transporter localized within the plasma membrane, lysosomes, early endosomes, and other vesicular compartments. It has not been associated with hypomyelination. Functional zinc flux assays in HeLa cells stably-expressing TMEM163 protein variants, L76R and L76P, revealed distinct attenuation or enhancement of zinc efflux, respectively. Experiments using a zebrafish model with knockdown of *tmem163a* and *tmem163b* (morphants) showed that loss of *tmem163* causes dysplasia of the larvae, locomotor disability and myelin deficit. Expression of human wild type *TMEM163* mRNAs in morphants rescues the phenotype, while the *TMEM163* L76P and L76R mutants aggravated the condition. Moreover, poor proliferation, elevated apoptosis of oligodendrocytes, and reduced oligodendrocytes and neurons were also observed in zebrafish morphants. Our findings suggest an unappreciated role for TMEM163 protein in myelin development and add *TMEM163* to a growing list of genes associated with hypomyelination leukodystrophy.

## 1. Introduction

Hypomyelinating leukodystrophies (HLDs) are a group of genetic disorders characterized by persistent deficit of myelin observed on magnetic resonance imaging (MRI) [1,2]. Patients with HLDs often present with nystagmus and axial hypotonia in early infancy, which gradually progress to ataxia and spasticity. Motor development is more affected than cognitive function. Alternatively, presentation later in life with a milder or normal clinical and radiological picture is also possible, and the latter form is considered transient hypomyelination in infancy [3,4,5]. Over the last ten years, comprehensive genetic testing such as whole exome sequencing (WES) or whole genome sequencing (WGS) has helped to identify novel genes associated with HLDs and reduce the number of unsolved cases [5,6,7,8]. To date, more than 20 genes have been associated with HLDs, which encode proteins involved in mRNA translation, heat-shock response, transcription factors, and proteins that are localized within the lysosomes and plasma membrane [9,10].

Using WES, we identified two unique missense variants located on the same DNA position within the *TMEM163* from two unrelated patients with HLD. Rodent *Tmem163* gene was first cloned in 2007 and initially named as synaptic vesicle 31 (SV31) [11]. Rodent TMEM163 protein is detected in plasma membrane, lysosomes, early endosomes, and other vesicular compartments [12]. Similarly, human TMEM163 heterologously expressed in cultured cells have plasma membrane and lysosomal localization [13]. RNA-seq study indicates the expression of *TMEM163* transcripts in oligodendrocytes [14]. Recently, one of our group proposed that TMEM163 likely belongs to the mammalian SLC30 (ZnT) family of zinc efflux transporters [15,16]. Recent Genome-Wide Association Study (GWAS) reports have implicated TMEM163 in Parkinson’s disease and diabetes; however, no mutations within *TMEM163* have been confirmed in these mono-genetic disorders. Here, we identified two *TMEM163* variants from two unrelated HLD patients and explored their phenotypic effects using functional assays on cultured cells and zebrafish model. We show for the first time that *TMEM163* plays a role in oligodendrocyte development and function, as well as being a novel gene associated with hypomyelination leukodystrophy.

## 2. Materials and Methods

### 2.1. Patients

The two families were identified at Peking University First Hospital (Beijing, China). This study was approved by the Medical Ethics Committee of Peking University First Hospital (No. [2005]-004). Written informed consent was secured from the patients’ guardians. Genomic DNA specimens were obtained from circulating leukocytes using standard procedures.

### 2.2. Whole Exome Sequencing (WES)

Whole exome sequencing (WES) was performed on family trios (proband, biological mother, and biological father) for both families. Exons were captured by SeqCap EZ MedExome Kit (Roche NimbleGen, Pleasanton, CA, USA) followed by sequencing on an Illumina X10 (2 × 150-nucleotide paired-end reads) by Joy Orient Translational Medicine Research Center Co., Ltd. Company (Beijing, China). WES data were comprehensively analyzed according to the in-house workflow previously described [17].

### 2.3. Cellular Zinc Flux Assays

HeLa cells were purchased from American Type Culture Collection (ATCC; Manassas, VA, USA). We selected HeLa cells because it does not express endogenous *TMEM163* transcripts (The Human Protein Atlas database, www.proteinatlas.org, accessed on 9 September 2019), and these cells adhere tightly on the plate culture well surface making it ideal for zinc flux assays that involve a lot of cell washing [15,18]. We performed site-directed mutagenesis using the wild-type *TMEM163* expression plasmid with mCherry fluorescent protein tag at the C-terminus. The recombinant TMEM163 protein with the mCherry fluorescent protein tag has been shown to be functional [15]. The DNA sequence integrity of all clones were verified by sequencing (Retrogen, San Diego, CA, USA). We then used HeLa cells to create stable cell lines that express wild-type TMEM163 and its variant proteins (L76P, L76R, D124A-D128A, and E286K). The TMEM163-D124-D128A is an inactive variant while TMEM163-E286K is a functional mutant [15]. We determined zinc efflux of wild-type TMEM163, TMEM163-L76P, TMEM163-L76R, TMEM163-D124-D128A, and TMEM163-E286K using the cell membrane permeant Newport Green fluorescent dye as previously described [15,18]. Detailed descriptions of methods are available in Appendix A.

### 2.4. In Vivo Assay

The wild-type zebrafish strain was Tübingen. Tg(mbp:GFP), Tg(oligo2:DRsred), and Tg(sox10:GFP) transgenic zebrafish were provided by China Zebrafish Resource Center, CZRC. Islet1:GFP and sox10:GFP stable transgenic lines were maintained by Qiang Wang’s laboratory. To obtain the zebrafish model with knockdown of *tmem163a* and *tmem163b* (morphant), embryos were injected with translation-blocking morpholinos (MOs) or guide RNA (gRNA) and Cas9 protein. *tmem163a*-MO, 5′-CATGCTGCTTTCCAACAGACACC-3′ and *tmem163b*-MO, 5′-CAGAGGAGGAGTCCGTCAT-3′ (herein referred to as *tmem163*-MO), were designed as complementary to *tmem163a* and *tmem163b* translation-blocking target. *tmem163a* gRNA1:5′-GGGAGATCCAGGACACCCAC-3′; *tmem163a* gRNA2: 5′-GGTGACGCTCATCCTGGCAG-3′; tmem163b gRNA1:5′-GGGGGCAGAAGGAGCGGGAC-3′; *tmem163b* gRNA2: 5′-GGTCTACAGGACCGGCCGTG-3′. sgRNAs were designed against the exon2 of *tmem163a* and exon1 of *tmem163b*, respectively. For the mutant and wild type (WT) *tmem163* model, the WT and mutant full-length coding sequence of human *TMEM163* (NM_030923.5) cloned into the pCS2-flag vector were injected into the yolk of zebrafish embryos at the one-cell stage at the indicated doses, respectively. For rescue of morphant phenotypes, MOs were injected into 2- to 4-cell embryos that had been injected with indicated doses of mRNA. The locomotor activity of zebrafish larvae at 120 hpf was tracked, recorded for 1 h, and then quantified with ZebraLab software (Viewpoint, Lyon, France). Myelination condition, number of oligodendrocytes, cell proliferation, and expression of interested protein were investigated by in situ hybridization (ISH) and immunofluorescence staining. Moreover, to comprehensively reflect the impact of *tmem163* deficiency on the transcriptome of zebrafish embryos, we collected 40–50 *tmem163* knockdown embryos and control MO (ctl-MO) injected embryos at 48 hpf and constructed RNA sequencing in parallel. RNA sequencing was performed by CapitalBio Technology (Beijing, China). Detailed descriptions of methods are available in Appendix A.

### 2.5. Statistics

All experiments were repeated at least three times with consistent results. All cell culture data were from biologic triplicates, unless otherwise indicated. Statistical analysis was performed with two-tailed, unpaired Student’s *t*-test for comparison of two groups, or ANOVA followed by Tukey’s post hoc test for multiple comparisons after verifying normality. Statistical analyses were performed using the GraphPad Prism 9.0 (San Diego, CA, USA) program. A *p*-value less than 0.05 was considered significant (* *p* < 0.05, ** *p* < 0.01, *** *p* < 0.001).

## 3. Results

### 3.1. Deleterious Variants of TMEM163 in Two Patients with Hypomyelination Leukodystrophy

**Figure 1:**
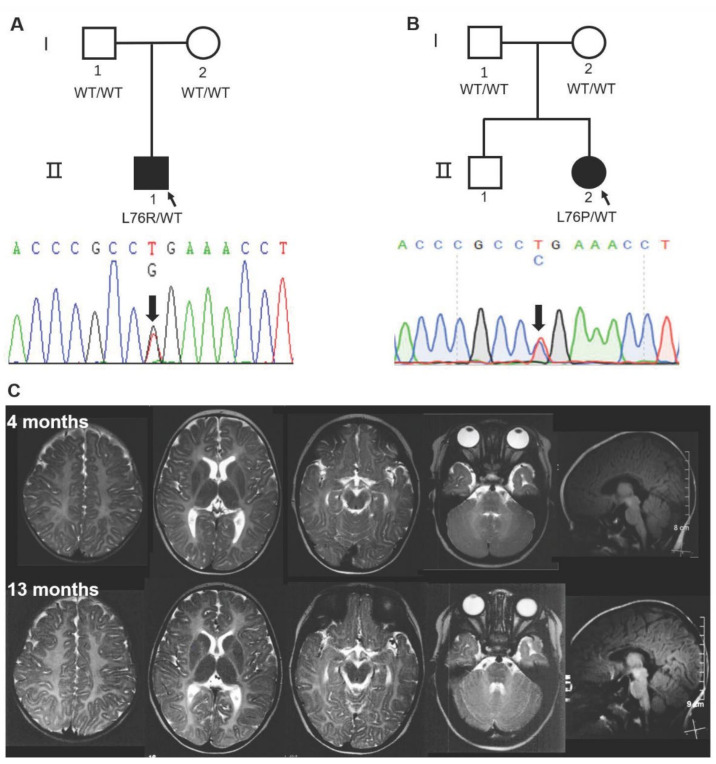
Sanger sequencing of two HLD pedigrees and representative MRI of Patient 2 with the *TMEM163*-L76P mutation. (**A**,**B**) De novo heterozygous c.227T>G p.(L76R) and c.227T>C p.(L76P) in *TMEM163* was identified in two probands, respectively. I, parents; II, progeny. (**C**) Diffuse hypo-intense signal on T2-weighted images at 4 months and 13 months indicated hypomyelination in cerebral white matter (**C**). WT, wild type.

Both patients were initially suspected of suffering from Pelizaeus-Merzbacher disease PMD or Pelizaeus-Merzbacher-like disease (PMLD) based on classical clinical presentation at an early age, including congenital nystagmus, motor delay, and myelin deficit on MRI (Figure 1, Table 1). Unexpectedly, the clinical prognosis of Patient 1 was favorable, with near-normalization of neurological signs. A limitation is a lack of MRI data at an older age for Patient 1, and it is unclear whether the myelin deficit resolved overtime as well. Patient 2 also improved gradually, but the course needs to be further explored as she grows up. To understand the natural history of the disease, additional monitoring of patients will be performed when possible. Further, a follow-up study will help uncover whether the myelin deficit on MRI for these patients resolved overtime, which may parallel what has been seen in patients with transient infantile hypomyelination associated with TMEM63A variants [3,4,5]. Detailed descriptions of each patient are available in Appendix A.

No promising variants in genes associated with PMD, PMLD, or other known HLDs subtypes were discovered. Interestingly, de novo heterozygous missense variants in Transmembrane 163 gene (*TMEM163*; NM_030923.5) stood out in two patients. The two variants, c.227T>G p.(L76R) in Patient 1 and c.227T>C p.(L76P) in Patient 2, shared the same nucleotide and amino acid residue site (Figure 1A,B). Both variants are very rare and are not present in the 1000 Genomes, ESP6500, gnomAD human population or Chinese 1000 Genomes databases. They are also predicted to be damaging by multiple prediction tools: MutationTaster, SIFT, Polyphen-2, M-Cap and LRT (Table S1). TMEM163 is a protein with 289 amino acids including six predicted transmembrane domains, and the mutated residue is highly conserved and localized within the cytoplasmic domain of the N-terminus region (Figure S1). Interestingly, the cytoplasmic domain of ZNT/SLC30 efflux transporters has been proposed to contain a zinc-sensing domain that facilitates zinc transport [19]. At the gene level, *TMEM163* has a relatively low residual variation intolerance score (RVIS) [−0.45 (24%)], a measure (ranking) of intolerance, which suggests that it is intolerant to functional genetic variation [20]. *TMEM163* has a positive missense Z-score of 1.65, which means it has fewer reported variants than expected and increased constraint (intolerance to variation) [21]. Additionally, *TMEM163* is predicted to have a low probability of being loss-of-function (LoF) intolerant (pLI) with a relatively low pLI score of 0.04 [21]. Using standard polymerase chain reaction (PCR) and real-time quantitative PCR, human *TMEM163* and mouse *Tmem163* transcripts have been reported in various tissues, notably in the brain, lung, pancreas, kidney, ovary, and testis [15,22]. RNA sequencing (RNA-seq) data from GTEx also shows *TMEM163* to be highly expressed in brain tissues, especially in the cerebellum [23]. RNA-seq data from cells revealed that *TMEM163* is expressed in human oligodendrocytes, and mouse *Tmem163* shows significantly higher expression in newly formed oligodendrocyte and myelinating oligodendrocytes (Brain RNA-seq database) (Figure S2) [14]. All of these findings suggest that *TMEM163* may be involved in the process of brain myelination. Combined with similar genotype and phenotype of the two patients, we propose *TMEM163* as a candidate gene for HLD.

### 3.2. Mutant TMEM163 Disrupted the Intracellular Zinc Homeostasis

**Figure 2:**
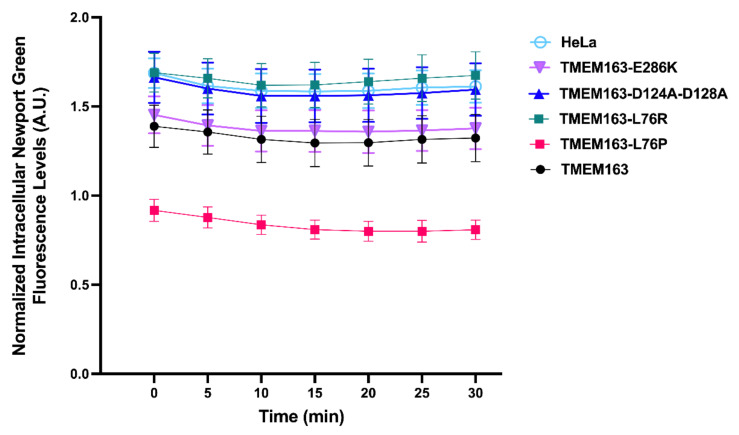
Intracellular zinc flux assay of HeLa cells stably expressing wild-type and variant TMEM163 proteins. The HLD-linked variant *TMEM163*-L76P showed significantly enhanced zinc efflux, while the *TMEM163*-L76R variant displayed marked loss of zinc efflux activity that mimicked the inactive *TMEM163*-D124A-D128A mutant. Unmodified HeLa cells and stable cells expressing the *TMEM163*-E286K mutant were included as additional controls. Significance testing was performed using ANOVA with repeated measures followed by a post-hoc analysis using Tukey’s multiple comparisons test (*p* < 0.0001, *n* = 4 independent trials). Data are represented as means ± SEM. A.U., arbitrary unit.

Recently, one of our groups reported that TMEM163 is a new member of the mammalian ZNT (SLC30) family of zinc efflux transporter, referred to as ZNT11 (SLC30A11), which is involved in cellular zinc homeostasis by extruding cytoplasmic zinc ions (Zn^2+^) to the extracellular milieu [15,16]. TMEM163 assembles into a functional dimer based on studies using its rodent counterpart [24]. Heterologously expressed TMEM163 localizes within the plasma membrane and membrane compartments such as the lysosomes and synaptic vesicles [11,12,15,22]. In our previous work, using human embryonic kidney (HEK)-293 cells that transiently expressed wild-type TMEM163 and certain non-synonymous single nucleotide variants of the protein (e.g., S61R, S95C, S193P and E286K), a significantly reduced zinc efflux has been documented for these reported variants compared with wild type [15]. These *TMEM163* variants were obtained from the single nucleotide polymorphism database (dbSNP, www.ncbi.nlm.nih.gov/snp, accessed on 9 September 2019) [15], which appear to be absent in control population databases except E286K with an allele frequency of 0.00001592 [21]. We also showed that mutating two aspartic acid residues of TMEM163 into alanine (i.e., D124A-D128A) at its putative zinc binding site [24] located within the second transmembrane domain produced an inactive protein [15]. To verify the mutational effect of the two new variants (L76R and L76P) identified from our patients, we determined intracellular zinc flux within stable cells expressing each protein variant along with positive and negative controls. HeLa cells stably expressing the L76R variant conferred a significant reduction of zinc efflux (*p* < 0.0001) that was comparable to the reported D124A-D128A inactive mutant [15] (Figure 2). On the other hand, HeLa cells stably expressing the L76P variant exhibited a significant increase in zinc efflux in comparison with stably expressing wild-type TMEM163 and control cells (*p* < 0.0001) (Figure 2). The differential effects on zinc efflux between L76P and L76R did not appear to be due to protein mis-localization since all stably expressing cell lines used in the zinc flux assay exhibited similar phenotypic features (Figure S3).

### 3.3. Loss of tmem163 in Zebrafish Causes Dysplasia of the Larvae, Locomotor Disability and Myelin Deficit

**Figure 3:**
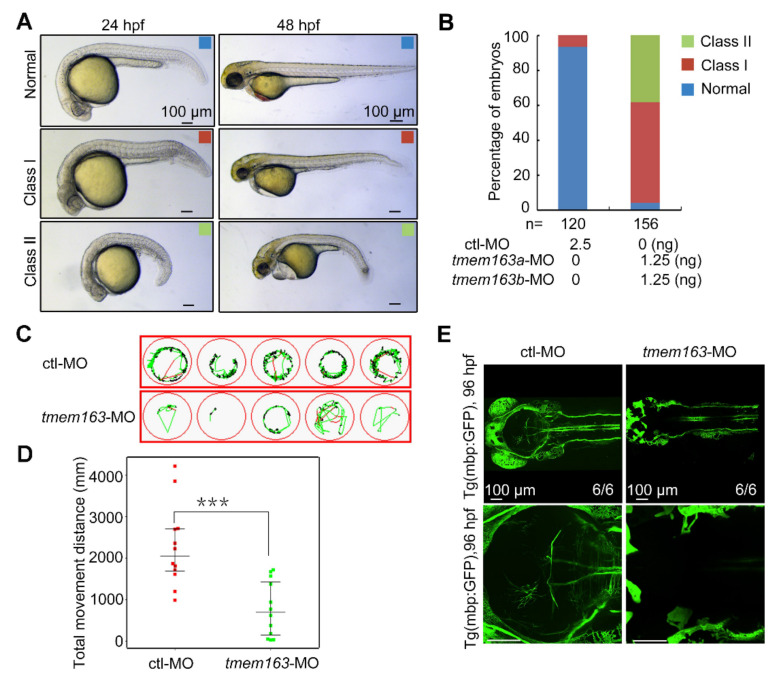
Impact of *tmem163* knockdown on morphology, locomotor ability and myelination in zebrafish larvae: (**A**) Representative images of the normal (blue), mild (red), and severe (green) phenotypes observed in the control and *tmem163*-MO injected groups at 24 hpf and 48 hpf. Scale bar: 100 μm. (**B**) Quantification of the percentage of normal, class I, and class II in two groups obtained. The percentages of embryos with each phenotype are shown in the bar graphs, and the number of embryos examined is listed under each bar. (**C**) Examples of swimming tracks of five single larvae of each condition shown in green at 120 hpf. (**D**) Scatter plot displaying the total movement distance by different larvae: MO-control (*n* = 12): 2689 ± 477 mm; tmem163-MO (*n* = 12): 748 ± 200 mm. *** *p* < 0.01 (Student’s *t*-test). (**E**) Representative pictures of the Tg(mbp:GFP) larvae injected with control and *tmem163*-MO at 96 hpf (dorsal views with anterior to the left). Scale bar: 100 μm.

Zebrafish is a popular model organism to underly the mechanisms of myelination in the central nervous system (CNS) in vivo [25] and it has been previously used to elucidate the possible pathogenesis of HLD19 in our previous collaborative research [8]. There are two zebrafish orthologs of human TMEM163 protein (NP_112185.1), tmem163a and tmem163b, respectively. Zebrafish Tmem163a and Tmem163b share 83% and 70% amino acid sequence identity with human TMEM163 protein, respectively, and in particular, their predicted transmembrane domains are highly conserved. Both *tmem163a* and *tmem163b* are zygotically expressed in zebrafish. At 24 h post-fertilization (hpf), the transcripts of *tmem163a* were mainly located in the CNS and Rohon-Beard (RB) sensory neurons when detected with whole mount in situ hybridization (WISH), while *tmem163b* is mainly expressed in the CNS and tail bud at 24 hpf. Both transcripts can be detected in brains and motor neurons of the spinal cord at 48 hpf (Figure S4A). To investigate the function of *tmem163* in zebrafish, we first explore the effect of *tmem163* deficiency on myelination in zebrafish embryos. Two morpholinos (MOs) for *tmem163a* or *tmem163b* mRNA were designed and synthesized to target sequences in the corresponding 5′ untranslated region (5′ UTR). The effectiveness of these morpholinos was confirmed by their ability to block the expression of the corresponding *tmem163* 5′ UTR-GFP fusion protein (Figure S4B). By microinjection of antisense morpholino oligonucleotides (MOs) into the yolk of 1-cell stage embryos to block mRNA translation, we got *tmem163* morphant zebrafish with knocked down *tmem163a* and *tmem163b*. Next, we found that the protein level of Tmem163 was reduced in embryos injected with *tmem163*-MOs compared with standard ctl-MO injected group detected with Western blot analysis (Figure S4C). This indicates that these MOs can block the translation of *tmem163* efficiently. Compared with ctl-MO injected embryos, embryos injected with 2.5 ng of *tmem163*-MOs exhibited severe disruption in the development of the CNS, characterized by the loss of midbrain-hindbrain boundary (MHB) structures and ventricles, as well as perturbed diencephalon and cerebellum at 24 hpf, followed by hydrocephalus at 48 hpf (Figure 3A,B). These results suggest that *tmem163* plays a role in CNS development. At 48 hpf, it was apparent that the dorso-ventral axis of the developing brain was also significantly reduced in the morphants. By 120–144 hpf, they did not survive due to stalled development, pericardial edema, and widespread tissue necrosis. Next, swimming ability of larvae at 120 hpf was analyzed by measuring the total movement distance. The total movement distance (in mm) of *tmem163* knockdown larvae was very significantly reduced (Figure 3C,D). To assess the function of *tmem163* in CNS myelination in vivo, we utilized a stable transgenic line mbp:EGFP, which labels myelinating oligodendrocytes under the control of the myelin basic protein (mbp) promoter [26]. Interestingly, EGFP signal was significantly reduced in *tmem163* morphants compared to control larvae (Figure 3E). To further confirm the association between developmental malformation observed in zebrafish and *tmem163* deficiency, we establish another *tmem163* model using mosaic crispant (Figure S5). Results showed that zebrafish embryos injected with *tmem163*-gRNAs presented with a significantly higher percentage of developmental malformation and myelin deficit (Figure S6), which is consistent with what has been observed in MO model. Altogether, these results indicate that loss of *tmem163* function in zebrafish embryos results in dysplasia of the larvae, locomotor disability and myelin deficit, which is consistent with the patients’ phenotype.

### 3.4. Mutant TMEM163 Causes Dysplasia of the Larvae and Myelin Deficit in Zebrafish

**Figure 4:**
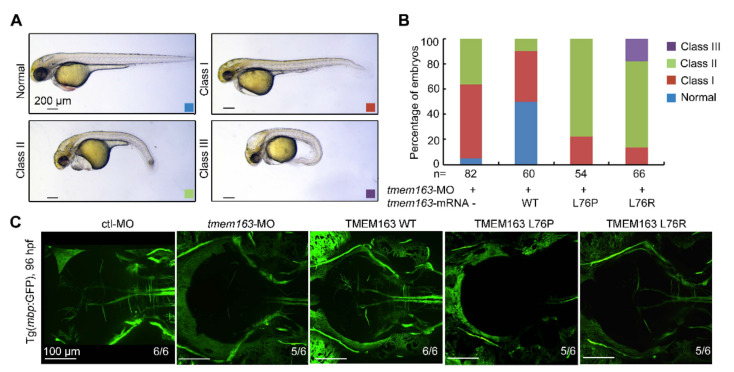
Functional analysis of *TMEM163* mutations in zebrafish injected with translation-blocking morpholinos: (**A**) Representative images of the normal (blue), mild (red), severe (green) and profound (purple) phenotypes observed in the *tmem163*-MO, MO+*TMEM163* WT, MO+*TMEM163* L76P, MO+*TMEM163* L76R injected groups at 48 hpf. Scale bar: 200 μm. (**B**) Percentage of living embryos showing any phenotype at 48 hpf. Co-injection of *tmem163*-MO with a human wild-type *TMEM163* mRNA partially rescued phenotypes of *tmem163* morphants such as degeneration of CNS, hydrocephalus, and bent tails, whereas injection of *TMEM163* mRNA bearing the p.L76P or p.L76R mutation failed to rescue the phenotype. The number of embryos examined is listed under each bar. (**C**) The myelination in CNS is disrupted. Illustration of the Tg(mbp:GFP) larvae injected with control-MO, *tmem163*-MO, *TMEM163* WT, *TMEM163* L76P, *TMEM163* L76R at 96 hpf (dorsal views with anterior to the left). Both the mutations disorganize the myelin in the brain. The ratios of affected embryos are indicated. Scale bar: 100 μm.

We employed *tmem163* knockdown embryos to test the functional consequences of the two *TMEM163* variants found in patients. When injected with an in vitro synthesized mRNA encoding the human wild-type *TMEM163*, 50% of the resulting embryos developed normally with no phenotype, demonstrating rescue effects and functional conservation between the human *TMEM163* and zebrafish *tmem163* genes (Figure 4A,B). Unexpectedly, injection of mRNAs harboring the human *TMEM163* mutations L76P or L76R resulted in a high percentage of embryos with more severe phenotype (see Table S2), suggesting that these mutations not only disrupted the ability of *TMEM163* to rescue but also exhibited a dominant negative mutation (Figure 4A,B). We also analyzed the effect of *TMEM163* L76P and L76R mutations in the formation of CNS myelin in zebrafish embryos. The myelination in zebrafish upon expression of mutant TMEM163 proteins showed myelin deficit similar to the *tmem163* morphants (Figure 4C).

### 3.5. Tmem163 Is Required for the Survival and Proliferation of Oligodendrocytes

**Figure 5:**
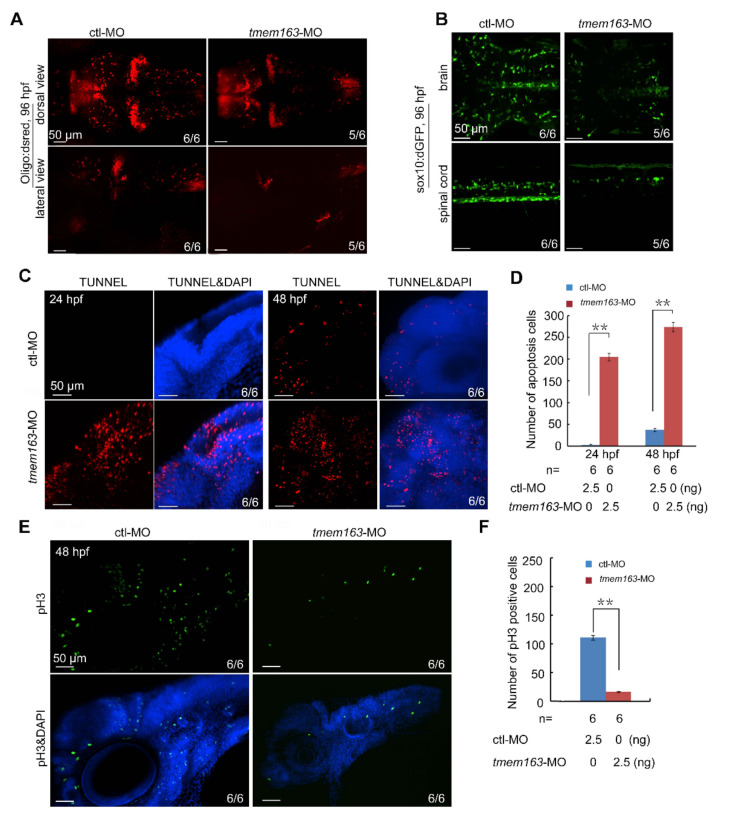
Loss of *tmem163* leads to reduction of oligodendrocytes: (**A**) Fluorescent express pattern of oligo2:DsRed in larvae injected with control and *tmem163*-MO at 96 hpf (anterior to the left). The ratio of embryos with representative morphology was shown in the right corner of each picture. Scale bar: 50 μm. (**B**) Fluorescent express pattern of sox10:GFP in larvae injected with control and *tmem163*-MO at 96 hpf (anterior to the left). The ratios of affected embryos are indicated. Scale bar: 50 μm. (**C**) TUNNEL assay in embryos injected with control and *tmem163*-MO at 24 hpf and 48 hpf. Lateral views with the dorsal side pointing to the top. The ratios of affected embryos are indicated. Note the distinct increase in *tmem163* morphants at the indicated stages. Scale bar: 50 μm. (**D**) Quantification of apoptosis cells (TUNEL-positive cells) in the hindbrain region calculated from six embryos. ** *p* < 0.01 (Student’s *t*-test). (**E**) Cell proliferation in *tmem163* morphants is impaired as revealed by immunofluorescent stained with anti-phosphorylated H3 antibody (green) and DAPI (blue). The ratio of embryos with representative signals was shown in the right corner of each picture. Scale bar: 50 μm. (**F**) Quantification of proliferative cells (GFP-positive cells) in the hindbrain region calculated from six embryos. ** *p* < 0.01 (Student’s *t*-test).

To explore the molecular mechanisms underlying hypomyelination associated with TMEM163, another transgenic zebrafish strain, oligo2:DsRed, were used to investigate the number of oligodendrocytes in *tmem163* morphants. It was found that down-regulation of *tmem163* reduced the number of oligodendrocytes dramatically at 96 hpf (Figure 5A). Similarly, the cell numbers of oligodendrocytes labelled with GFP were also reduced in *tmem163* knockdown embryos compared with control animals (Figure 5B). In order to uncover the cellular defects responsible for the reduction of oligodendrocytes induced by *tmem163* deficiency, TUNNEL assay was performed. Results showed that the number of TUNNEL-positive apoptotic cells in the head region of *tmem163* morphants was far more than that in the control group at 24 hpf and 48 hpf (Figure 5C,D). We then investigated cell proliferation by phospho-Histone 3 (pH3) immunostaining at 48 hpf. Embryos injected with *tmem163*-MOs had less pH3-positive cells in the head regions (Figure 5D,E), being indicative of impaired cell proliferation. Overall, loss of *tmem163* in zebrafish embryos promotes cell apoptosis and hinders cell proliferation, thereby leading to a reduction of the oligodendrocyte number and eventually myelin deficit.

### 3.6. Tmem163 Is Required for the Development of Neuron

To comprehensively reflect the impact of *tmem163* deficiency on the transcriptome of zebrafish embryos, we collected 40–50 *tmem163* knockdown embryos and control MO injected embryos at 48 hpf and constructed RNA sequencing in parallel. The gene expression data were analyzed for significance. In total, we identified 1108 significantly differentially expressed genes (DEGs, FC > 2, *p*-value < 0.05); 512 were upregulated and 596 were downregulated (Figure S7A). Gene Ontology (GO) analysis showed that the top biological processes included neurogenesis, nervous system development, generation of neurons and system development (Figure S7B). These data suggest that *tmem163* plays important roles in the development of neurons. To verify this in silico observation, we analyzed the development of neurons in huc:GFP transgenic zebrafish embryos. At 48 hpf, the number of neurons expressing GFP in *tmem163*-deficient embryos was reduced compared to control MO injected embryos (Figure S7C). These data suggest that *tmem163* is required for the development of neurons.

## 4. Discussion

Here we present two patients with HLD, which are associated with *TMEM163* variants. Both patients were initially suspected of suffering from PMD or PMLD based on classical clinical presentation at an early age, including congenital nystagmus, motor delay, and myelin deficit on MRI. Unexpectedly, the clinical prognosis of Patient 1 was favorable, with near-normalization of neurological signs. A limitation is a lack of MRI data at an older age for Patient 1, and it is unclear whether the myelin deficit resolved overtime as well. Patient 2 also improved gradually, but the course needs to be further explored as she grows up. To understand the natural history of the disease, additional monitoring of patients will be performed when possible. Further, a follow-up study will help uncover whether the myelin deficit on MRI for these patients resolved overtime, which may parallel what has been seen in patients with transient infantile hypomyelination associated with TMEM63A variants [3,4,5]. Based on the current genomic evidence, the two similar rare deleterious variants, L76R and L76P in *TMEM163*, are likely responsible for the clinical manifestation observed in the two patients.

Single nucleotide polymorphisms (SNPs) in *TMEM163* have been reported to be associated with an increased risk of Parkinson’s disease and diabetes [13,27,28,29]. Additionally, the expression of TMEM163 was found reduced in patients with Mucolipidosis type IV [22] or HPS [30]. However, no deleterious variants in *TMEM163* have been reported as causes of a Mendelian disease. Here, we first propose *TMEM163* as a disease-causing gene of a neurologic disease, hypomyelination leukodystrophy.

*TMEM163* is located on chromosome 2q21.3 and was first identified in rat brain tissue as a novel synaptic vesicle membrane protein and initially named SV31 (synaptic vesicle membrane protein of 31 kDa) in 2007 [11]. Subsequently, TMEM163 was further revealed to be a zinc transporter, which forms stable dimers in artificial lipid nanodiscs [12,22,24]. Recently, one of our groups showed that TMEM163 protein extrudes cytoplasmic Zn^2+^ to the extracellular milieu, and certain non-synonymous single nucleotide variants of the protein (e.g., S61R, S95C, S193P and E286K) reduced cytoplasmic zinc efflux [15]. In the current study, stable expression of L76R in HeLa cells led to significant reduction of zinc efflux that was comparable to the reported D124A-D128A inactive mutant [15], suggesting that L76R results in a loss-of-function mutation. On the other hand, stable expression of L76P mutation confers a gain-of-function phenotype that markedly increased zinc efflux from HeLa cells. Both leucine and proline are nonpolar and hydrophobic amino acids. The hydrophobicity of proline is weaker than that of leucine due to its shorter side chain. Arginine is a basic (positively charged) and hydrophilic amino acid. Thus, substitution of either amino acid residue at position 76 might have produced a negative effect on the structure and stability of TMEM163 protein. The loss-of-function phenotype caused by L76R partially mimicked that of the less-active E286K protein variant [15], which could be explained by the fact that both R and K amino acid residues are positively charged and could interfere with ionic or side-chain interactions within the protein structure. On the other hand, proline is known to potentially kink protein structure and has been shown to produce gain-of-function phenotype in certain proteins such as that observed in the Mucolipin-3 ion channel [31]. Overall, both L76R and L76P disrupted the intracellular zinc homeostasis in cells from different direction. It is not clear, however, how changes in zinc homeostasis leads to myelin deficit as observed in our patients.

Zinc is enriched in the brain, but is tightly regulated. Zinc deficiency or zinc excess can rapidly lead to brain cell death via necrotic, apoptotic, or autophagic pathways [32,33]. Indeed, it has been shown that increased intracellular zinc leads to oligodendrocyte death in multiple injury paradigms [34,35], but reduction of zinc levels has also been shown to contribute to glutamate-induced oligodendrocyte excitotoxicity [36]. To date, the role of *TMEM163* in glial cells or white matter is unknown. Nevertheless, our findings suggest a previously unappreciated role for zinc in neuronal myelination. By searching the gene in the RNA-seq database, we confirm the expression of *TMEM163* in oligodendrocyte, the myelinating cell in the CNS. Next, to further explore the role of *TMEM163* in myelination, functional studies were performed using a zebrafish model. Consistent with the patients’ phenotype, zebrafish with reduced levels of tmem163 display a higher percentage of abnormal morphology, impaired motor ability and myelination, suggesting an essential role for tmem163 in neurogenesis and myelination. Similar results have been observed in another zebrafish model with loss of *DEGS1*, a novel disease-causing gene of HLDs identified recently [8]. Compared with wild type, the expression of mutant *TMEM163*, L76R or L76P, could not only rescue the phenotype of *tmem163*-MO larvae but aggravated the condition. The group with L76R or L76P also showed myelin deficiency like *tmem163*-MO. These results confirmed the pathogenicity of the variants and association between mutation in *TMEM163* and HLD disease phenotype. The RNA-seq data also lend credence to the essential role of *TMEM163* in CNS development. It is interesting to note that *Tmem163* KO mice have been generated by Salm et al. in 2020 [37]. The investigators reported that *Tmem163* KO mice were viable and fertile, and that the loss of *Tmem163* affected the ATP-evoked behavior leading to a reduction in peak amplitude of ATP-evoked currents in dorsal root ganglion (DRG) neurons [37]. While the Tmem163 KO phenotype appears to affect DRG neurons, Salm et al. did not report additional CNS-related phenotype, presumably because they did not examine potential subtle deficits in motor function of the *Tmem163* KO mice, and in particular, motor dysfunction caused by abnormal myelination. Nevertheless, it is possible that an abnormal phenotype in *Tmem163* KO mice may be detectable at a particular developmental stage. As we have observed in two patients, the symptoms were severe only in early childhood. The transient clinical symptoms may be explained by the redundancy of zinc efflux transporters in human and mouse cells, such that other zinc effluxers may rescue the phenotype deficiency as the organism matures.

Multiple factors can lead to myelin deficit, which can be attributed to the decrease in proliferation and differentiation or the increase in apoptosis of oligodendrocyte. In zebrafish with loss of tmem163, reduction of oligodendrocyte numbers, increased apoptosis, and impaired cell proliferation were all noted. Accordingly, enhanced apoptosis and inhibition of proliferation of the oligodendrocyte may be the underlying cause of disease phenotype in the animal. Further experiments are needed to confirm if cell differentiation is also affected and to determine the precise mechanism underlying the abnormal cell proliferation and apoptosis within the zebrafish *tmem163* morphants.

## 5. Conclusions

Although molecular mechanisms underlying hypomyelination in patients or Tg zebrafish remain largely unknown, our findings showed that zebrafish tmem163 is critical for myelination in vivo, and mutant TMEM163 protein disrupted intracellular zinc homeostasis in cultured human cells. Overall, this work adds *TMEM163* to the list of genes associated with HLDs.

## Figures and Tables

**Table 1:** Clinical characteristics of two patients with variants in *TMEM163*.

Patient	1	2
Mutation	L76R	L76P
Gender	male	female
Age	7 years and 3 months	3 years and 3 months
Nystagmus		
Age at onset	after birth	2 months
Age resolved	8 months	2 years
Development		
Walking without support	2 years	2 years and 10 months
Language development	mild delay	mild delay
Myelin deficit (age)	7 months	4 month, 13 months
Findings at last neurological examination	hypotonia	hypotonia
Other	abnormal VEP ^1^	not available

^1^ Visual evoked potentials.

## Data Availability

The data presented in this study are available on request from the corresponding author. The data are not publicly available due to privacy.

## Supplementary Materials

The following materials are available online at https://www.mdpi.com/article/10.3390/cells11081285/s1. Figure S1: Predicted TMEM163 protein structure and variant conservation; Figure S2: Expression of *TMEM163* in oligodendrocyte of human and mouse brain tissues; Figure S3: Representative fluorescence micrographs of stable cell lines expressing wild-type TMEM163 and protein variants; Figure S4. Expression of pinhead during zebrafish embryogenesis and the efficiency detection of *tmem163*-MO; Figure S5: Generation of tmem163 crispants using CRISPR/Cas9 technology; Figure S6: Impact of tmem163 knockdown on morphology, locomotor ability and myelination in zebrafish larvae injected with gRNA; Figure S7: tmem163 is required in neurogenesis; Table S1: Characteristics of *TMEM163* gene variants; Table S2: Data of the rescue experiment.

## Appendix A

### Appendix A.1. Case Report

Patient 1, 7 years old, is a male of Chinese descent born at term by elective caesarean section after an uneventful pregnancy (Figure 1A). His birth weight was 4200 g. Horizontal nystagmus was noted after birth and resolved by 8 months. His motor milestones were mildly delayed (age in month when achieved): head control (6), roll over (7), grasp objects (10), sit alone (12), aided stand (20), and walk alone with abnormal gait (24). His language and mental ability were relatively spared. He could smile socially at 2 months, fear stronger at 5 months and started to speak at 12 months. In 2011, he was first evaluated in the clinic at 7 months of age and low axial muscle tone was observed. Brain MRI showed delayed myelination in white matter that appeared like that of a newborn. Blood chemical tests, inborn metabolic error screening, fundus examination, electroencephalogram and visual evoked potentials were all normal. Abnormal brain-stem auditory evoked potentials were observed. PMD was suspected clinically, but no sequence variants or duplication of PLP1 were detected by Sanger sequencing and multiplex ligation-dependent probe amplification (MLPA). The boy has improved gradually, and at the age 7 years, he attends regular school with an average performance, except for slow and abnormal gait when running and walking down. Brain MRI examination was not performed again, which precluded further analysis of myelin development. No seizure, abnormal hearing, sight, or behavior were noted in the course.

Patient 2 is a girl age 3 years 3 months of Chinese descent. She is the second child of an unrelated Chinese couple (Figure 1B), and was born at term with a birth weight of 3489 g. No complications were noted during the pregnancy and delivery. At the age of 2 months, horizontal nystagmus was observed, which resolved by the end of the second year of life. In 2017, she was first seen in clinic at 4 months, where besides nystagmus, delayed development accompanied with low axial muscle tone were noted. Her head circumference was in the 50th percentile at that time. Her delayed developmental milestones became obvious overtime (age in month when achieved): head control (3), roll over (10), sit alone (14), walk alone with abnormal gait (34), and speak (24). At the age of 3 years and 3 months, she could say several complete sentences and sing a whole nursery rhyme by herself, albeit with unclear pronunciation. Assessments performed at 13 months using the Chinese versions of Gesell Development demonstrated moderate impairment in the gross motor domain. The fine motor domain, speech and language domain, personal and social domain and adaptive domain were all mildly impaired. When examined at the age of 15 months, axial hypotonia was noted, while physical growth parameters, muscle strength, hearing and sight were all normal. No regression, seizure, or abnormal behavior were noted. Diffuse myelin deficit in the brain on MRI was first noted at age 4 months and no improvement was observed when examined at 13 months again (Figure 1C). Screening of liver function, kidney function, electrolyte, lactic acid, homocysteine, vitamin B12, inborn metabolic error and activity of lysosomal enzyme revealed nothing. Pelizaeus-Merzbacher-like disease (PMLD) was suspected clinically.

### Appendix A.2. Methods

#### Appendix A.2.1. Cellular Zinc Flux Assays

HeLa cells were maintained in antibiotic-free Dulbecco’s Modified Eagle’s Media (DMEM; 4.50 g/L glucose, 0.58 g/L L-glutamine, 0.11 g/L sodium pyruvate; Corning) supplemented with 10% fetal bovine serum (FBS; Thermo Scientific, Waltham, MA, USA). The cells were placed in a humidified 37 °C incubator supplied with 5% CO_2_. The cells were seeded in quadruplicate wells of a 96-well culture plate coated with poly-D-lysine. Twenty-four hours post-seeding, the cells were incubated with zinc chloride (100 μM) and the zinc ionophore, pyrithione (10 μM) before exposure to Newport Green. HeLa cells stably expressing the L76P variant grew much slower than the other stable cell lines and unmodified HeLa cells. Thus, we adjusted the number of the L76P cell line seeded in the culture plate to take this phenotypic difference into account for each experiment. A kinetic run every minute for a 30-min period was performed and fluorescence reads from five-minute intervals were obtained for data analysis (*n* ≥ 4 independent trials). The relative fluorescence units were background-subtracted and normalized using averaged total cell counts.

#### Appendix A.2.2. Zebrafish Strain

Zebrafish embryos were obtained by mating of adult fish using standard methods. All fish strains were maintained individually as inbred lines. The wild-type zebrafish strain was Tubigen. *Tg(mbp:GFP)*, *Tg(oligo2:DRsred)*, and *Tg(sox10:GFP)* transgenic zebrafish were provided by China Zebrafish Resource Center, CZRC. *Islet1:GFP* and *sox10:GFP* stable transgenic lines were maintained by Qiang Wang’s lab. Embryos were reared at 28.5 °C until processing for analyses at desired stages. All zebrafish experiments were in strict accordance with the Regulations for the Care and Use of Laboratory Animals as published by the Ministry of Science and Technology of China, and the Institute of Zoology’s Guidelines for the Care and Use of Laboratory Animals.

#### Appendix A.2.3. ISH and Immunofluorescence Staining

Embryos were fixed in 4% paraformaldehyde (PFA) at desired stages. Hybridization was performed at 65 °C with digoxigenin-labelled RNA probes. The antisense digoxigenin-labelled RNA probe for zebrafish *tmem163a* (ENSDARG00000079858, primers: forward 5′- ATGCGCCTGAAGCCTCATGA -3′; reverse, 5′- CTAATACGACTCACTATAGGGTGTTACTCAAAGCGCTCATAGTT -3′) and *tmem163b* (ENSDARG00000088227, primers: forward 5′- ATGACGGACTCCTCCTCTGC -3′; reverse, 5′- GCTAATACGACTCACTATAGGGTGCTACTCGAAGCGCTCGTAGTGT -3′) were amplified by PCR and generated by T7 RNA Polymerase in vitro transcription using the DIG-RNA Labelling Kit (Roche). Embryos were collected at the desired stage, fixed in 4% paraformaldehyde overnight, washed with PBS containing 0.1% Tween 20 for 30 min, blocked with 1% bovine serum albumin for 1 h at room temperature, and then incubated with anti-phospho-H3 (3377, Cell Signaling Technology) for 24 h at 4 °C. All immunofluorescence images were captured using a Nikon A1R+ confocal microscope with the same settings for all samples within each experiment. For fluorescence microscopy of cultured HeLa cells stably expressing wild type and mutant TMEM163 proteins, representative images were taken using an Olympus IX-71 microscope. Monochromatic RGB images of the cells were captured using the CellSens software version 1.18 (Olympus America, Center Valley, PA, USA). We then used Adobe Photoshop CC 2021 (Adobe Systems, San Jose, CA, USA) to represent the mCherry fluorescence in red colour.

#### Appendix A.2.4. Antisense MO and mRNA Injections

For MO knockdowns, embryos were injected with translation-blocking MOs obtained from GeneTools LLC. MO-*tmem163a*, 5′- CATGCTGCTTTCCAACAGACACC-3′ and MO-*tmem163b*, 5′- CAGAGGAGGAGTCCGTCAT -3′ (herein referred to as MO-*tmem163*), was designed as complementary to *tmem163a* and *temem163b* translation-blocking target. Briefly, 1nl of mixture with 1.25 ng MO-*tmem163a* and 1.25 ng MO-*tmem163b* or ctl-MO (5’-CCT CTT ACC TCA GTT ACA ATT TAT A 3’) was injected into the yolk of 1-cell-stage embryos (herein referred to as *tmem163*-MO). After injection, embryos were incubated at 28.5 °C until the desired stage was reached. In order to verify the efficiency of the MOs, proteins were extracted from 50 embryos in each group at 24 hpf, and conducted with Western Blot analysis with anti-TMEM163 antibody (ab76783, Abcam).

To verify the specificity of *tmem163* MO, the 5’UTR of *tmem163a* and *tmem163b* was amplified and subcloned into pEGFP-N3 vectors to form a fusion protein Tmem163 5’UTR-GFP. The plasmids were injected into embryos at the 1-cell stage with control MO or *tmem163* MOs. The fluorescent images were photographed at the late gastrulation stage.

To analyse the function of mutant TMEM163 protein, the wild-type and mutant full-length coding sequence of human *TMEM163* (NM_030923.5) were amplified and cloned into the pCS2-flag vector by Beijing SYKM Gene Biotechnology Co., Ltd. (Beijing, China) Capped mRNAs were synthesized with the mMessage mMachine kit (Ambion) and purified with RNeasy mini kit (Qiagen) according to the manufacturers’ instructions. Individual mRNA was injected into the yolk of zebrafish embryos at the one-cell stage at the indicated doses, and the phonotype of embryos were observed and photographed. For rescue of morphant phenotypes, MOs were injected into 2- to 4-cell embryos that had been injected with indicate doses of mRNA.

#### Appendix A.2.5. Larval Locomotor Behavior Assay

By 120 hpf, zebrafish larvae perform spontaneous swimming and their visual system is fully developed. Zebrafish larvae were transferred to 48-well microplates, with one larva per well. The zebrafish was allowed to habituate for 10 min in the chamber of an automated tracking device called a ZebraBox (Viewpoint, Lyon, France). The locomotor activity was tracked, recorded for 1 h, and then quantified with ZebraLab software (Viewpoint, Lyon, France). All tracking experiments were performed at least in triplicate. All measures were averaged across larvae within each group (*n* = 12 larvae/group) and are reported as population means ± SD.

#### Appendix A.2.6. Generation of Zebrafish F0 Crispant by CRISPR/Cas9

Guide RNA(gRNA) target sequences were selected using the CHOPCHOP online tool v1 (*tmem163a* gRNA1:5’- GGGAGATCCAGGACACCCAC -3’; *tmem163a* gRNA2: 5’- GGTGACGCTCATCCTGGCAG-3’; *tmem163b* gRNA1:5’- GGGGGCAGAAGGAGCGGGAC -3’; *tmem163b* gRNA2: 5’- GGTCTACAGGACCGGCCGTG -3’;). sgRNAs were designed against the exon2 of *tmem163a* and exon1 of *tmem163b*, respectively. The gRNAs were synthesized in vitro with T7 RIBOMAX express large scale RNA production system (Promega), purified with Rneasy mini kit (Qiagen) and coinjected with Cas9 protein (M0646, New England BioLabs, Ipswich, MA, USA) into Tg(mbp:GFP) embryos at the 1-cell stage. For testing of the CRISPR efficiency, the genomic regions surrounding gRNA-targeted sequences were amplified by PCR with the following primers: for *tmem163a* crispants, 5′- CCCTAGGCTCAATGCCTGTC -3′ (forward) and 5′- ACACACCCTAACTGCCACTTT -3’ (reverse); for *tmem163b* crispants, 5′- TAAGTTGGCGGTTTGTTCCG -3′ (forward) and 5′- TCCAAAAAGCAGAGATGCCAAC -3′ (reverse). The amplified DNA fragment was purified and digested with T7 endonuclease I (M0302, New England BioLabs) for 30 min at 37 °C, which recognizes and cleaves non-stringently matched DNA then subjected to electrophoresis with 2% agarose gel.

#### Appendix A.2.7. Live Imaging

Zebrafish embryos at the desired stage were embedded in the 1% low melting-point agarose and placed in a temperature- controlled sample holder. Live-images were captured under Andor high-speed spinning disk confocal Dragonfly (Belfast, UK).

#### Appendix A.2.8. RNA Isolation, cDNA Library Preparation and Sequencing

RNA sequencing was performed by CapitalBio Technology (Beijing, China). Briefly, 40–50 pooled embryos for each group harvested at 48 hpf and were snap-frozen in liquid nitrogen immediately and stored at −80 °C. Embryos total RNA was extracted with Trizol (Invitrogen) and assessed with Agilent 2100 BioAnalyzer (Agilent Technologies, Santa Clara, CA, USA) and Qubit Fluorometer (Invitrogen). Total RNA samples that meet the following requirements were used in subsequent experiments: RNA integrity number (RIN) > 7.0 and a 28S:18S ratio > 1.8.RNA-seq libraries were generated and sequenced by CapitalBio Technology (Beijing, China). The triplicate samples of all assays were constructed an independent library, and do the following sequencing and analysis. The NEB Next Ultra RNA Library Prep Kit for Illumina (NEB) was used to construct the libraries for sequencing. NEB Next Poly(A) mRNA Magnetic Isolation Module (NEB) kit was used to enrich the poly(A) tailed mRNA molecules from 1 μg total RNA. The mRNA was fragmented into ~200 base pair pieces. The first-strand cDNA was synthesized from the mRNA fragments reverse transcriptase and random hexamer primers, and then the second-strand cDNA was synthesized using DNA polymerase I and RNase H. The end of the cDNA fragment was subjected to an end repair process that included the addition of a single “A” base, followed by ligation of the adapters. Products were purified and enriched by polymerase chain reaction (PCR) to amplify the library DNA. The final libraries were quantified using KAPA Library Quantification kit (KAPA Biosystems, Cape Town, South Africa) and an Agilent 2100 Bioanalyzer. After quantitative reverse transcription-polymerase chain reaction (RT-qPCR) validation, libraries were subjected to paired-end sequencing with pair end 150-base pair reading length on an Illumina HiSeq sequencer (Illumina).

#### Appendix A.2.9. RNA-Seq: Data Analysis

The genome of human genome version of hg38 was used as reference. The sequencing quality were assessed with FastQC (v0.11.5) [38] and then low quality data were filtered using NGSQC (v2.3.3) [39].The clean reads were then aligned to the reference genome using HISAT2 (v2.1.0) with default parameters. The processed reads from each sample were aligned using HISAT2 against the reference genome. The gene expression analyses were performed with StringTie (v1.3.3b) [40]. DESeq (v1.28.0) [41] was used to analyse the DEGs between samples. Thousands of independent statistical hypothesis testing was conducted on DEGs, separately. Then a *p*-value was obtained, which was corrected by using the false-discovery rate (FDR) method, and the corrected *p*-value (q-value) was calculated by correcting using Benjamini-Hochberg method. The *p*-value or q-value were used to conduct significance analysis. Parameters for classifying significantly DEGs are ≥2-fold differences (|log2FC| ≥ 1, FC: the fold change of expressions) in the transcript abundance and *p* ≤ 0.05.

The annotation of the DEGs were performed based on the information obtained from the database of ENSEMBL, NCBI, Uniport, GO, and KEGG.
