# Supplementary material for: Functional Study of TMEM163 Gene Variants Associated with Hypomyelination Leukodystrophy
Source: Cells. 2022 Apr 9;11(8):1285. doi: 10.3390/cells11081285 (PMC9031525; doi:10.3390/cells11081285)
Supplement: Supplementary file 1 [file cells-11-01285-s001.zip › cells-1540883-supplement.pdf]

Functional Study of *TMEM163* Gene Variants Associated with Hypomyelination Leukodystrophy

Huifang Yan[1,2,3,4,†], Shuyan Yang[5,†], Yiming Hou[6], Saima Ali[7], Adrian Escobar[7], Kai Gao[1], Ruoyu Duan[1], Thomas Kubisiak[4], Junyu Wang[1], Yu Zhang[1], Jiangxi Xiao[8], Yuwu Jiang[1,3,9], Ting Zhang[5], Ye Wu[1,3], Margit Burmeister[4,10], Qiang Wang[6,11], Math P. Cuajungco[7,12,*], and Jingmin Wang[1,2,3,9,*]

1 Department of Pediatrics, Peking University First Hospital, Beijing, 100034, China; yanhuifang96@bjmu.edu.cn (H.Y.); gaokaipku@hsc.pku.edu.cn (K.G.); ruoyuduan123@pku.edu.cn (R.D.); wjy-pediatrics@bjmu.edu.cn (J.W.); zhangyunihao@bjmu.edu.cn (Y.Z.); jiangyuwu@bjmu.edu.cn (Y.J.); dryewu@bjmu.edu.cn (Y.W.)
2 Joint International Research Center of Translational and Clinical Research, Beijing, 100191, China
3 Beijing Key Laboratory of Molecular Diagnosis and Study on Pediatric Genetic Diseases, Beijing, 100034, China
4 Michigan Neuroscience Institute, University of Michigan, Ann Arbor, MI, 48109, USA; kubisiat@umich.edu (T.K.); margit@umich.edu (M.B.)
5 Beijing Municipal Key Laboratory of Child Development and Nutriomics, Capital Institute of Pediatrics, Beijing, 100020, China; shuyanyang79@126.com (S.Y.); zhangtingcv@126.com (T.Z.)
6 State Key Laboratory of Membrane Biology, Institute of Zoology, University of Chinese Academy of Sciences, Chinese Academy of Sciences, Beijing, 100101, China; houyiming@ioz.ac.cn (Y.H.); qiangwang@ioz.ac.cn (Q.W.)
7 Department of Biological Science, California State University, Fullerton, CA, 92831, USA; saimaali@fullerton.edu (S.A.); adrian_escobar@csu.fullerton.edu (A.E.)
8 Department of Radiology, Peking University First Hospital, Beijing, 100034, China; xiaojiangxi@bjmu.edu.cn
9 Key Laboratory for Neuroscience, Ministry of Education/National Health and Family Planning Commission, Peking University, Beijing, 100191, China
10 Departments of Computational Medicine & Bioinformatics, Psychiatry and Human Genetics, University of Michigan, Ann Arbor, MI, 48109, USA
11 Institute for Stem Cell and Regeneration, Chinese Academy of Sciences, Beijing, 100101, China.
12 Center for Applied Biotechnology Studies, California State University, Fullerton, CA, 92831, USA
† These authors contributed equally to this work.

Correspondence: JW: wangjingmin@bjmu.edu.cn, Department of Pediatrics, Peking University First Hospital, No.1 Xi'an Men Street, West District, Beijing 100034, China; MPC: mcuajungco@fullerton.edu, Biological Science, California State University Fullerton, 800 North State College Blvd., Fullerton, CA, 92831, USA.

Supplemental Data

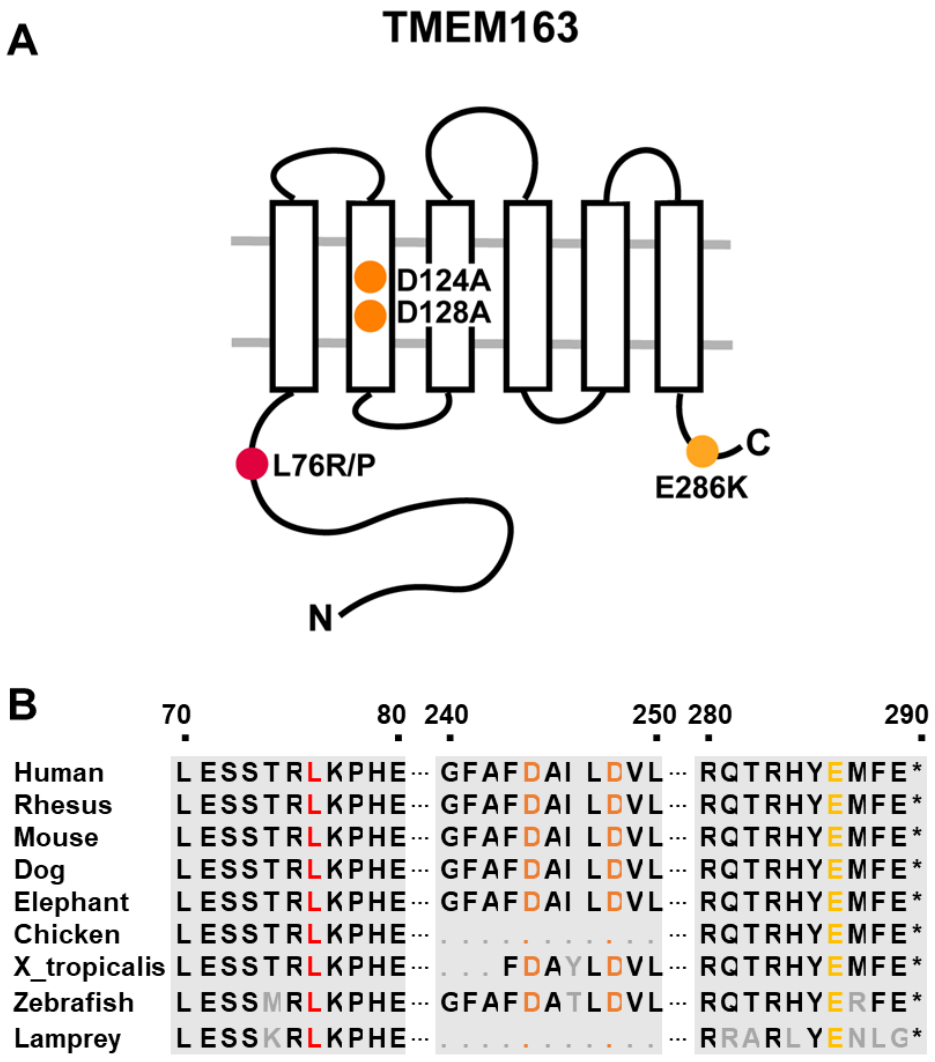

Figure S1. Predicted TMEM163 protein structure and variant conservation. (**A**) Schematic drawing of the six putative transmembrane domains of TMEM163 protein. The Leucine at position 76 substituted with either Arginine (L76R) or Proline (L76P) is on the cytoplasmic N-terminus region (red circle). The Glutamic acid to Lysine substitution (E286K) is on the C-terminus region. Two Aspartic acid residues at positions 124 and 128 are located within the second transmembrane domain and identified as zinc-binding region that becomes inactivated upon alanine substitution (D124A-D128A). (**B**) Clustal alignment of TMEM163 protein from nine vertebrate species. The residues altered in the presented individuals are highlighted in red.

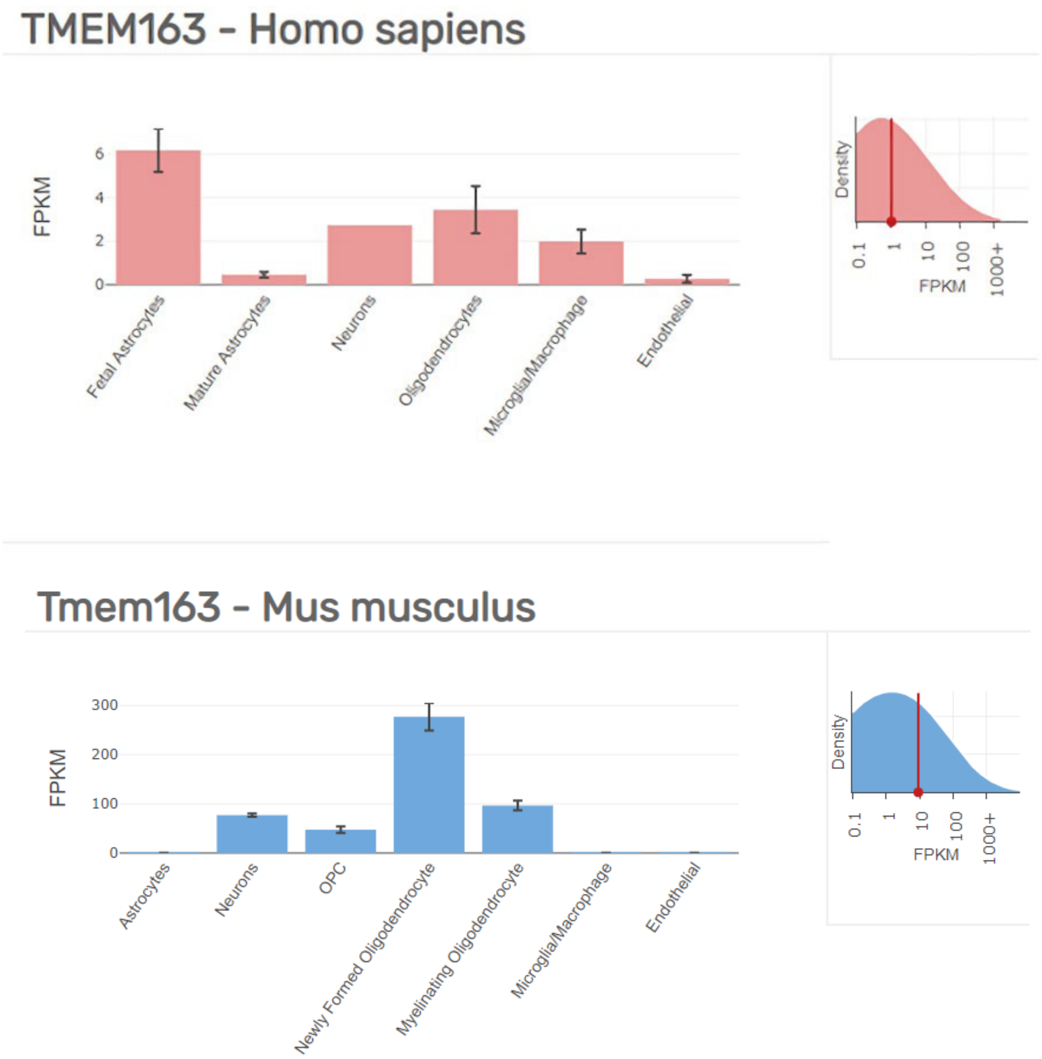

Figure S2. Expression of TMEM163 genes in oligodendrocyte of human and mouse brain tissues. Data obtained from Brain RNA-seq database (http://www.brainrnaseq.org).

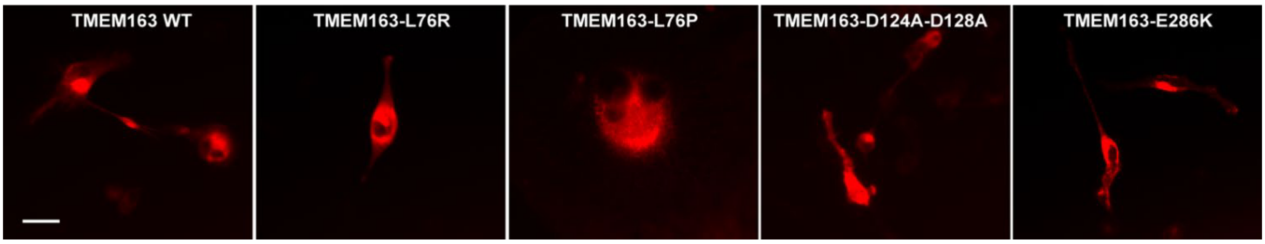

Figure S3. Representative fluorescence micrographs of stable cell lines expressing wild-type and mutant TMEM163 proteins tagged with mCherry fluorescent protein. Scale bar: 50 μm.

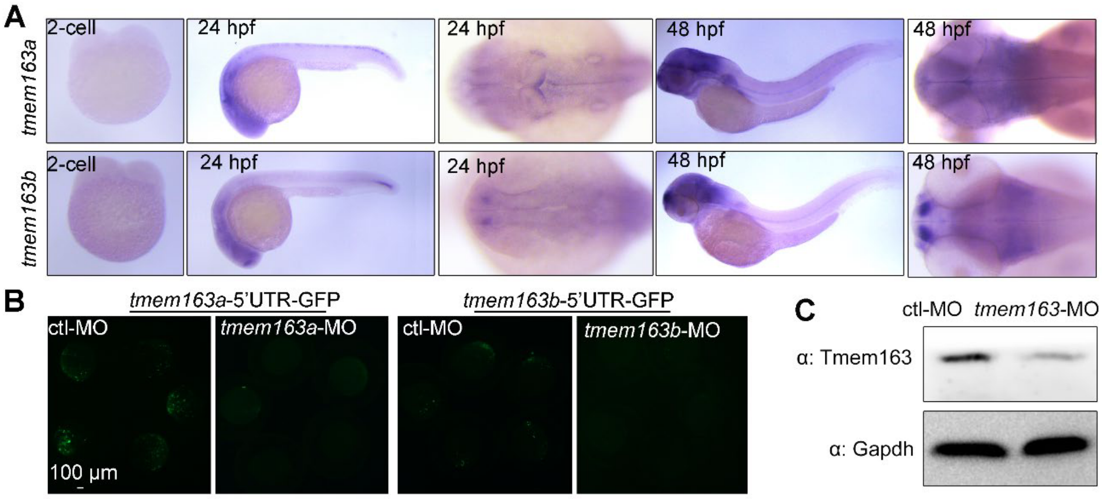

Figure S4. Expression of pinhead during zebrafish embryogenesis and the efficiency detection of *tmem163* MO. (**A**) Expression of *tmem163a* and *tmem163b* in wild-type embryos was analyzed by whole mount in situ hybridization at the indicated stages. Scale bars: 50 μm. (**B**) Effectiveness of tmem163-MOs. Note that the GFP expression was inhibited by specific morpholinos but not ctl-MO. (**C**) Western blot of total lysates from embryos injected with *tmem163*-MO and ctl-MO with anti-TMEM163 antibody at 24 hpf.

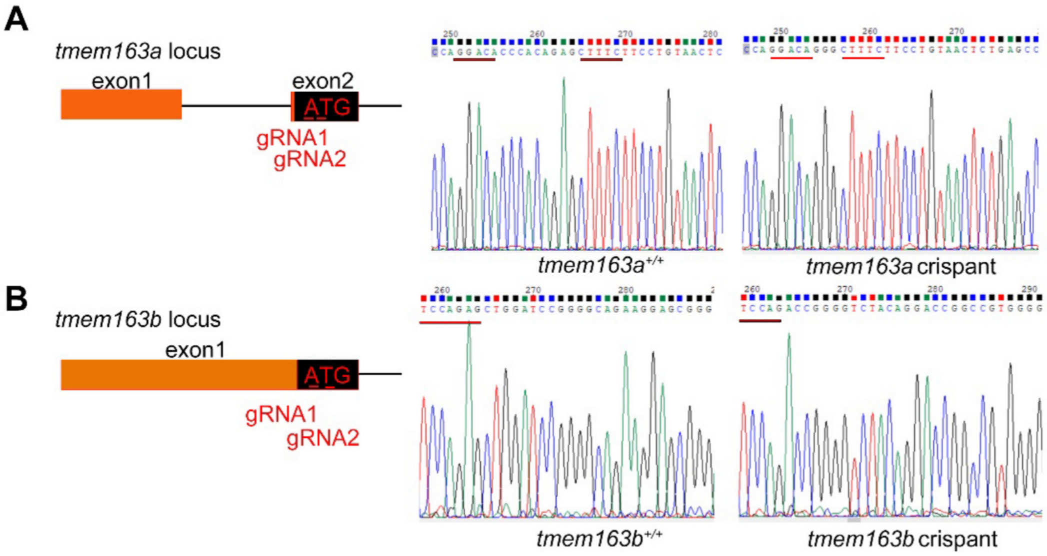

Figure S5. Generation of tmem163 crispants using CRISPR/Cas9 system. Location of the sgRNAs target sites are exhibited, respectively. The sequence of tmem163a (**A**) or tmem163b (**B**) wild-type and mutant is listed.

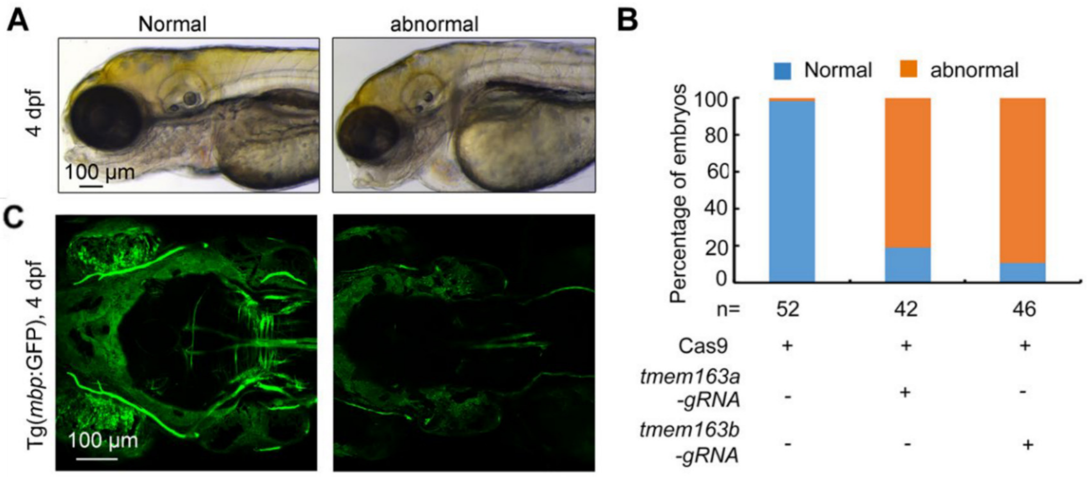

Figure S6. Defects of morphology and myelination in *tmem163* crispants. (**A**) Representative images of the normal and abnormal phenotypes observed in the control and *tmem163*-gRNA injected groups 4 dpf. Scale bar: 100 μm. (**B**) Quantification of the percentage of normal and abnormal in *tmem163* crispant embryos. The percentages of embryos with each phenotype are shown in the bar graphs, and the number of embryos examined is listed under each bar. (**C**) Representative pictures of the Tg(mbp:GFP) larvae injected with control and *tmem163*-gRNA at 96 hpf (dorsal views with anterior to the left). Scale bar: 100 μm.

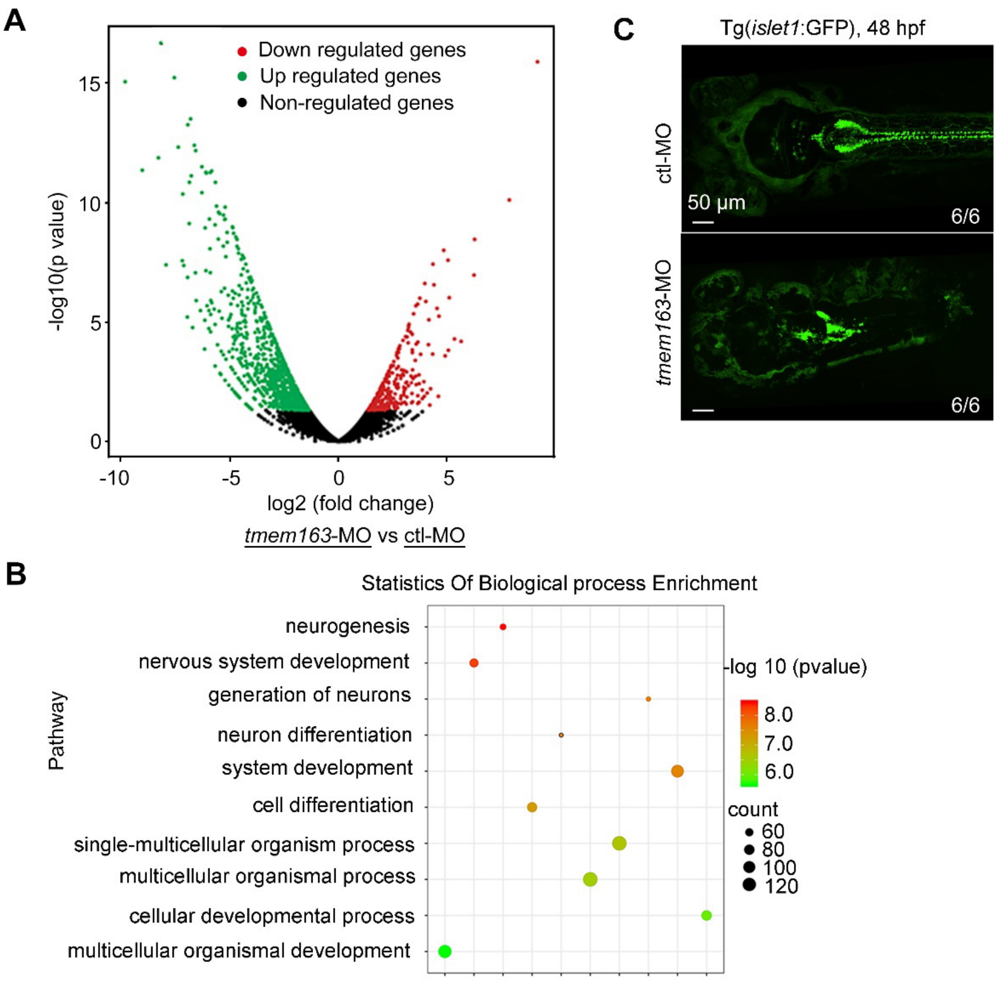

Figure S7. tmem163 is required in neurogenesis. (**A**) Volcano plots display the differentially expressed genes in the tmem163 morphants at 48 hpf. (**B**) The top ten gene ontology categories of the tmem163 knockdown transcripts that are significantly altered. (log2 fold change) > 1, q-value < 0.001). (**C**) Dorsal view of a stable Tg(islet1:EGFP) zebrafish at 48 hpf. The ratio of embryos with representative expression pattern was shown in the right corner of each picture. Scale bar: 50 μm.

Table S1. Characteristics of *TMEM163* gene variants

Pt	Gene	CDS	Protein	MAF	GERP	CADD	REVEL	SI	Po	MT	LRT	MC
1	*TMEM163*	c.227T>G	p.(L76R)	-	5.1	26.5	0.91	D	D	D	D	D
2	*TMEM163*	c.227T>C	p.(L76P)	-	5.1	27.1	0.81	D	D	D	D	D

Note: Reference sequences of *TMEM163* is NM_030923.4. - indicates the variant absent in population database including 1000G, ExAC or gnomAD; SI: SIFT, Po: Polyphen-2, MT: MutationTaster, MC:M-Cap, GERP : GERP++_RS.

Table S2. Data of the rescue experiment.

Phenotype	ctl-MO	*tmem163*-MO	*tmem163*-MO +*TMEM163* WT	*tmem163*-MO +*TMEM*163 L76P	*tmem163*-MO +*TMEM163* L76R
**Normal**	95%	5%	50%	0	0
**class I**	5%	55%	40%	18%	10%
**class II**	0	37%	10%	72%	70%
**Class III**	0	0	0	0	20%

Note: ctl, control; MO, morpholino; WT, wild type.
